# microRNA-Mediated Messenger RNA Deadenylation Contributes to Translational Repression in Mammalian Cells

**DOI:** 10.1371/journal.pone.0006783

**Published:** 2009-08-27

**Authors:** Traude H. Beilharz, David T. Humphreys, Jennifer L. Clancy, Rolf Thermann, David I. K. Martin, Matthias W. Hentze, Thomas Preiss

**Affiliations:** 1 Molecular Genetics Division, Victor Chang Cardiac Research Institute, Sydney, New South Wales, Australia; 2 School of Biotechnology & Biomolecular Sciences and St Vincent's Clinical School, University of New South Wales, Sydney, New South Wales, Australia; 3 European Molecular Biology Laboratory, Heidelberg, Baden-Württemberg, Germany; University College London, United Kingdom

## Abstract

Animal microRNAs (miRNAs) typically regulate gene expression by binding to partially complementary target sites in the 3′ untranslated region (UTR) of messenger RNA (mRNA) reducing its translation and stability. They also commonly induce shortening of the mRNA 3′ poly(A) tail, which contributes to their mRNA decay promoting function. The relationship between miRNA-mediated deadenylation and translational repression has been less clear. Using transfection of reporter constructs carrying three imperfectly matching let-7 target sites in the 3′ UTR into mammalian cells we observe rapid target mRNA deadenylation that precedes measureable translational repression by endogenous let-7 miRNA. Depleting cells of the argonaute co-factors RCK or TNRC6A can impair let-7-mediated repression despite ongoing mRNA deadenylation, indicating that deadenylation alone is not sufficient to effect full repression. Nevertheless, the magnitude of translational repression by let-7 is diminished when the target reporter lacks a poly(A) tail. Employing an antisense strategy to block deadenylation of target mRNA with poly(A) tail also partially impairs translational repression. On the one hand, these experiments confirm that tail removal by deadenylation is not strictly required for translational repression. On the other hand they show directly that deadenylation can augment miRNA-mediated translational repression in mammalian cells beyond stimulating mRNA decay. Taken together with published work, these results suggest a dual role of deadenylation in miRNA function: it contributes to translational repression as well as mRNA decay and is thus critically involved in establishing the quantitatively appropriate physiological response to miRNAs.

## Introduction

miRNAs are ∼22 nucleotide (nt) sized gene regulators that have pervasive roles in development and disease [1], [2]. They interact with argonaute (Ago) proteins [3] and guide RNA-induced silencing complexes (RISC or miRNP) to target mRNAs [1], [2]. Binding of animal miRNAs to imperfectly matching sequences, usually in the 3′ untranslated region (UTR) of their target mRNAs [4], can inhibit accumulation of the encoded proteins by reducing mRNA translation and/or stability [5], [6], [7], [8]. A single miRNA may regulate the expression of hundreds of proteins, with the expression of targets often only mildly attenuated [9], [10]. All four human Ago-subfamily proteins are capable of functioning in miRNA-mediated repression [11] and interact with similar sets of mRNAs and protein partners, notably the three GW182 paralogs, TNRC6-A, -B, and C [12], which function downstream of the Ago proteins in the miRNA mechanism [13], [14], [15], [16], [17]. To explain the translation inhibitory action of miRNAs, both initiation [14], [18], [19], [20], [21], [22], [23], [24], [25], [26], [27], [28] and post-initiation based models [27], [29], [30], [31], [32], [33], [34] have been proposed and the matter is subject to active debate [5], [6], [7], [35], [36], [37]. It has also been observed that miRNAs accelerate deadenylation of their target mRNAs in *Drosophila melanogaster*, zebrafish and mammalian systems, which contributes to miRNA-mediated destabilisation of their targets [28], [38], [39], [40], [41], [42].

We reported previously that a synthetic miRNA termed miCXCR4 [31] inhibits translation initiation of a *Renilla* luciferase (R-luc) reporter mRNA in transfected HeLa cells [19]. Specifically, we saw that translation initiation driven solely by the cap structure or solely by the poly(A) tail was partially responsive to miCXCR4, while only an mRNA carrying both end modifications was subject to the full extent of translational repression [19]. Our work concurred with several other studies either *in vivo* [18] or *in vitro* [20], [23], [25], [26], [28], indicating that the function of the mRNA 5′ m^7^GpppN cap structure and the cap-binding eukaryotic initiation factor (eIF) 4E was impaired during miRNA-mediated repression. Our observations furthermore suggested that a miRNA inhibits poly(A) tail function during translation initiation [19], possibly by interfering with the formation of an mRNA closed-loop configuration, which is enabled by joint binding of eIF4E and the poly(A)-binding protein PABP to the bridging protein eIF4G [43], [44], [45]. In further support of this model, a poly(A) tail was required to demonstrate miRNA-mediated repression in two mammalian *in vitro* translation systems [20], [25]. By contrast, a study examining repression by the endogenous let-7 miRNA family in HeLa cells reported no poly(A) tail dependence of translational repression [18]. Moreover, several reporter mRNAs lacking a poly(A) tail could still be silenced in mammalian and *D. melanogaster* cells, though in some cases at a reduced level [40], [42], [46]. Thus an emerging consensus is that the presence of a poly(A) tail on a target mRNA and/or its removal by deadenylation is not strictly required for miRNA-mediated translational repression, but to what extent it may still augment it is less clear.

This situation prompted us to further investigate the involvement of the poly(A) tail and its removal by deadenylation in miRNA-mediated translational repression. We report here that an established model of let-7-mediated repression in mammalian cells [18] features marked deadenylation that precedes the onset of observable translational repression and mRNA destabilisation. We find that depletion of argonaute co-factors can reduce let-7-mediated repression without noticeably affecting mRNA deadenylation, confirming that repression can be enacted by a deadenylation-independent mechanism. Nevertheless, we show that the magnitude of translational repression is reduced when the let-7-targeted reporter mRNA lacks a poly(A) tail, or when we block the deadenylation of a reporter mRNA with poly(A) tail. Our observations thus indicate that in mammalian cells deadenylation can contribute not only to mRNA decay but also translational repression mediated by an endogenous miRNA.

## Results

### Let-7 promotes target mRNA deadenylation

**Figure 1:**
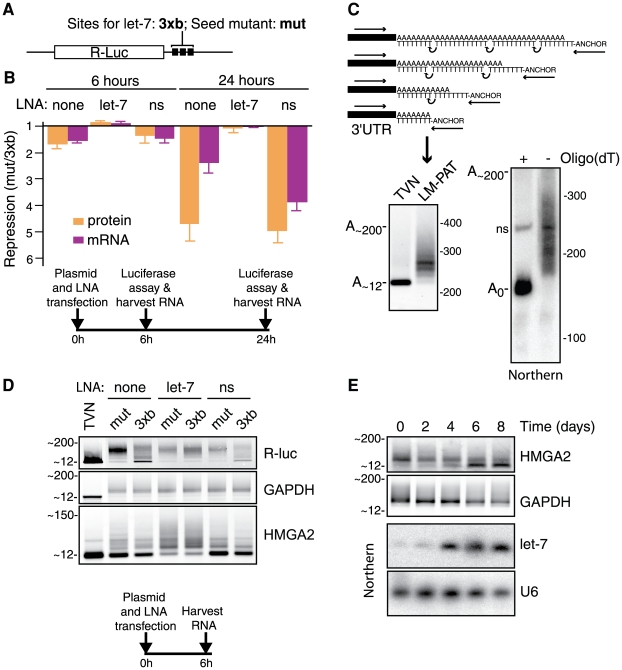
mRNAs targeted by let-7 exhibit shortened poly(A) tails. (*A*) Schematic of R-luc constructs carrying three let-7 target sites in their 3′ UTR (functional: R-luc-3xbulge; seed region mutated: R-luc-3xbulgemut [18]). (*B*) Let-7-dependent repression of R-luc protein and mRNA levels. HeLa cells were cotransfected with plasmids pR-luc-3xb or pR-luc-mut and pGL3 (F-luc) as control, and with an LNA anti-miR to let-7, miR-499 (non-specific; ns), or mock transfected (none) as indicated. Protein and RNA was extracted from cells at 6 and 24 hours after transfection. R-luc expression was normalised to F-luc expression for both protein and mRNA, measured by luciferase assay and qPCR, respectively. Repression by endogenous let-7 was calculated as the ratio of R-luc-mut expression to that of the corresponding R-luc-3xb. The bars represent averages of 2–3 measurements with standard error (24 hour protein) or range (all others). (*C*) Schematic of the LM-PAT assay and performance test. An LM-PAT assay for GAPDH mRNA was performed with HeLa cell total RNA (left panel). To mark the position of the shortest possible LM-PAT product, PCR was also carried out with cDNA primed with an anchor-(dT)_12_VN oligonucleotide that clamps to the 3′UTR–poly(A) junction (labeled ‘TVN’ above lane). An RNA sample was also subjected to RH-Hs GAPDH oligonucleotide-mediated cleavage by RNAse H and high resolution northern blot analysis (right panel). Cleavage was further done in the presence of oligo(dT) to generate a 3′ UTR fragment without poly(A) tail. Position of size markers are indicated to the right of the panels (in base-pairs or nucleotides, respectively). (*D*) The poly(A) tail lengths of R-luc reporter and endogenous *GAPDH* (control) or *HMGA2* mRNAs were measured by LM-PAT assay six hours after transfection. (*E*) P19 cells were induced to differentiate by treatment with retinoic acid and RNA was purified from cells at the times indicated. Let-7 and U6 RNA (loading control) expression were measured by northern blotting (10 µg total RNA per lane), while *HMGA2* and *GAPDH* mRNA poly(A) tail lengths were measured by LM-PAT assay.

**Figure 2:**
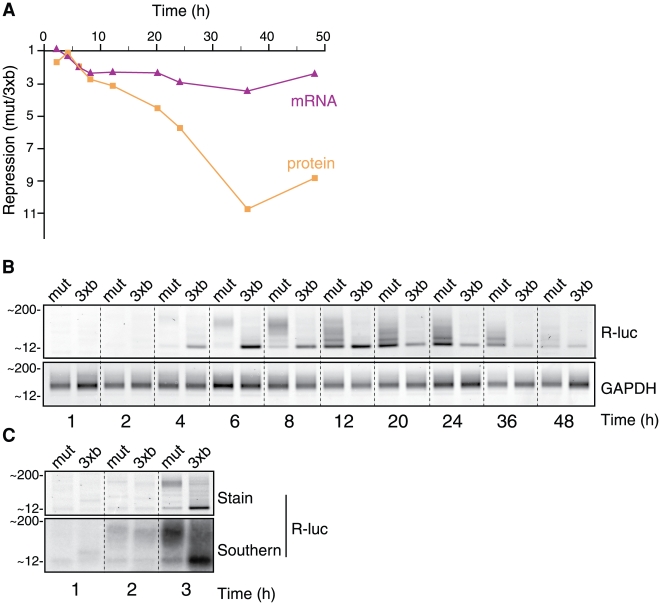
Let-7-triggered target mRNA deadenylation is rapid. HeLa cells were transiently transfected with let-7 reporter plasmids as in Fig. 1 , followed by protein and RNA analysis at the indicated times. (*A*) Let-7-mediated repression on protein (squares) and mRNA (triangles) levels, calculated as in Fig. 1B. Results are averages of triplicate data and are comparable to results gained from two independent experiments. See Fig. S2 for raw expression data. (*B*) LM-PAT assays of R-luc-3xb and R-luc-mut mRNAs (GAPDH as control). (*C*) LM-PAT products were visualized by ethidium bromide stain (top) or Southern blot (bottom).

To further characterise the role of target mRNA deadenylation in the miRNA mechanism we employed an established model of let-7-mediated repression in mammalian cells [18]. Thus, we transfected HeLa cells with the plasmid pRL-3xbulge expressing a Renilla luciferase (R-luc-3xb) mRNA with three bulged let-7 sites in the 3′UTR region, together with the plasmid pGL3 expressing a Firefly luciferase mRNA (F-luc) as a transfection efficiency control. Parallel transfection of cells with the seed region-mutated plasmid pRL-3xbulgemut provided non-targeted R-luc expression levels as a reference. A schematic of the R-luc mRNAs is shown in Fig. 1A (see Materials & Methods for further plasmid details). Reporter protein and mRNA levels in transfected cells were measured by dual luciferase assay and real-time RT-PCR, respectively. After normalisation against the F-luc transfection efficiency control, repression by endogenous let-7 was calculated as the expression ratio of non-targeted reference (R-luc-mut) over let-7-target (R-luc-3xb). This demonstrated that let-7 repressed the R-luc-3xb reporter on both protein and mRNA levels (∼5-fold and ∼2.5-fold respectively, 24 hours after transfection; Fig. 1B). Note that repression of R-luc protein level as defined here is an aggregate of changes in mRNA stability as well as translation efficiency. The component of repression on the translational level accounts for the stronger reduction of protein than mRNA levels and is apparent at 24 hours, becoming more evident at later time points (see Fig. 2A).

In parallel, we also measured miRNA-mediated deadenylation by the RT-PCR-based ligation-mediated poly(A) test (LM-PAT) [47], [48]. We chose the LM-PAT assay because of its higher sensitivity compared to high resolution Northern blotting. Initial tests with the abundant GAPDH mRNA showed that the LM-PAT assay measures poly(A) tail lengths with an accuracy similar to high resolution northern blotting (Fig. 1C, see also Supporting Information S1). The LM-PAT analysis revealed that six hours post-transfection R-luc-mut mRNA had a long poly(A) tail (intensity peak at ∼150 adenosines), while the R-luc-3xb mRNA presents with markedly shortened tails (Fig. 1D, top panel). To control for LM-PAT assay performance we further analysed the endogenous *GAPDH* mRNA, which we found to display an intermediate and invariant poly(A) tail length (intensity peak at ∼50 nt) throughout (Fig. 1D, middle panel). Note that the GAPDH result reflects tail-length distribution in a steady-state mRNA population. By contrast, the R-luc plasmid transfection is characterised by a much more transient expression of reporter mRNA (see also Fig. 2 *and* Fig. S2). As an additional specificity control we also co-transfected cells with a locked nucleic acid (LNA) anti-miR to let-7 or an LNA against the irrelevant miR-499 as the non-specific (ns) control anti-miR. This showed that the let-7 anti-miR restores R-luc-3xb reporter expression to R-luc-mut levels on the protein as well as mRNA level (Fig. 1B). The let-7 anti-miR further selectively prevented R-luc-3xb mRNA deadenylation (Fig 1D). Collectively, these results show that this reporter system features let-7-specific translational repression, as well as moderate mRNA destabilisation as previously reported [18]. Importantly, they further show that it also displays let-7-mediated mRNA deadenylation.

### Endogenous miRNA-targeted mRNAs accumulate with short poly(A) tails

We then asked if an endogenous let-7-targeted mRNA similarly undergoes deadenylation. We thus performed LM-PAT assays for *HMGA2* mRNA, a confirmed let-7 target [49], [50]. We found in HeLa cells that *HMGA2* mRNA is present primarily in an oligo-adenylated form, while transfection of let-7 anti-miR shifts it to a longer-tailed form (≥40 nt, Fig. 1D); this is blocked by actinomycin D treatment suggesting that the longer-tailed *HMGA2* mRNA molecules arise from new transcription (data not shown). Conversely, we observed a shift from longer-tailed to oligo-adenylated *HMGA2* during neuronal differentiation of mouse embryonic carcinoma P19 cells (Fig. 1E). We chose this cellular transition because it is known to entail a dramatic increase in let-7 levels (Fig. 1E) [51]. mRNA deadenylation is emerging as a feature of miRNA action in different systems [38], [39], [40], [41]. To extend observations in mammalian cells, we assessed the steady-state poly(A) tail length of seven validated miRNA targets [52], [53] in cell lines reported to express both the mRNAs and cognate miRNAs [54]. Six of these mRNAs clearly accumulated oligo-adenylated forms, while the ACTB control mRNA does not (see Supporting Information S1 & Fig. S1). This cursory screen of additional endogenous miRNA targets is thus consistent with miRNA-mediated deadenylation being widespread in mammalian cells. This mirrors conclusions from recent microarray analyses in *D. melanogaster*, which showed that a widespread miRNA-mediated destabilisation of target mRNAs was dependent on the CCR4:NOT deadenylase complex [42]. Importantly, our data demonstrate let-7-dependent deadenylation of the cancer-related *HMGA2* mRNA during a physiologically relevant cellular differentiation process as well as in the experimental conditions employed for our R-luc reporter studies.

### Let-7-mediated mRNA deadenylation is rapid

Next we explored the timing of miRNA-mediated deadenylation in relation to miRNA-directed translational repression, by performing a 48-hour time-course in HeLa cells transiently transfected with the let-7 reporter plasmids. While not being a strict pulse-chase scenario, this approach still featured a marked and transient burst of mRNA expression and likely mirrors the repression by a miRNA over the lifespan of an mRNA (see Fig. S2 for raw expression data). Between four and six hours, modest repression (∼2 fold) of both R-luc protein and mRNA levels becomes apparent (Fig. 2A). Beyond 8 hours the levels of the let-7-regulated mRNA stabilize at ∼2 to 3-fold less than control, but measured repression on the protein level continues to increase, reaching its maximal level (∼11-fold) at 36 hours, an increase that cannot be attributed to mRNA stability decreases alone. LM-PAT assays were performed in parallel (Fig. 2B, C). As expected, R-luc-mut mRNA accumulates as long-tailed molecules (∼150 nt of poly(A); up to eight hours) that become gradually deadenylated over time. Strikingly, while polyadenylated R-luc-3xb mRNA is initially detectable, it is already severely deadenylated three hours after transfection (Fig. 2C). These experiments indicate that miRNA-mediated deadenylation is a rapid step in the mechanism of miRNA action.

### Let-7-mediated repression can be attenuated despite ongoing deadenylation

**Figure 3:**
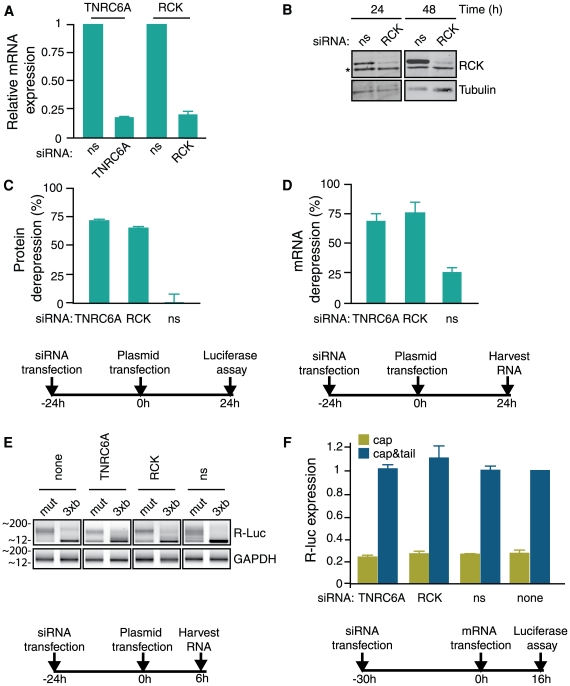
let-7-mediated repression is relieved by depletion of RCK/p54 or TNRC6A despite target mRNA deadenylation. HeLa cells were transfected with siRNAs targeting *GW182*, *RCK*, the non-specific miCXCR4 control (ns), or mock transfected (none) as indicated. (*A*) Residual mRNA levels were measured by qPCR between 24 and 48 hours and normalised against *RPL13a*. Bars represent the average expression of the relevant mRNA in three independent experiments with standard error. mRNA expression in cells transfected with ns was set at 1 (hence do not carry an associated error). (*B*) Extracts from cells harvested 24 or 48 hours after RCK or control siRNA transfection were analysed by western blotting and infrared fluorescent imaging using antibodies against RCK and tubulin. RCK protein level (normalised to tubulin loading control) was reduced to ∼25% (24 hours) and ∼11% (48 hours). * Cross-reacting non-specific band. (*C, D*) After 24 hours of knockdown, cells were transfected with let-7 reporter plasmids. R-luc activity (*C*) and mRNA levels (*D*) were measured 24 hours later and repression calculated as in Fig. 1. Derepression achieved by siRNA knockdown was calculated as 1 - (repression in siRNA treated cells/repression in “none” cells) and normalised to derepression by let-7 anti-miR seen in parallel transfections (set to 100% derepression, Fig. 1B. Bars represent averages of 4–10 (a) or 4–5 (b) independent experiments with standard error. (*E*) HeLa cells were transfected as above and total RNA purified 6 hours after plasmid transfection for LM-PAT assay of the R-luc and *GAPDH* mRNAs. Data representative of 3–4 independent experiments are shown. (*F*) After 30 hours of knockdown, cells were transfected with *in vitro* transcribed capped R-luc-mut mRNA with or without a poly(A) tail (expression of R-luc-mut with poly(A) tail in “none” set to 1.0 for each experimental set prior to averaging across experiments). Averaged results from 3–7 independent experiments are shown with standard error.

We furthermore depleted HeLa cells of the Ago co-repressors RCK/p54 or GW182 to explore the interdependence of let-7-mediated repression and deadenylation. We used proven siRNAs against RCK/p54 or the TNRC6A paralog of GW182 [55], which led to efficient knock-down, while miCXCR4 [19], [31] served as a non-targeting siRNA control in these experiments (Fig. 3A, B). Twenty-four hours after siRNA treatment, cells were transfected with let-7 reporter plasmids to measure effects on repression and deadenylation. The dual transfection regimen led to some variability in achievable repression in the non-specific control, as observed in previous reports [22], [55]. Similar to these studies, we scaled the data within each set to the mock transfection (0% derepression, equivalent to an average reduction of ∼2.5-fold on mRNA and ∼5-fold on protein level), and let-7 anti-miR controls served as a reference for 100% derepression (see Fig. 1B), to account for this systemic variation in the set-point of repression. RCK or TNRC6A depletion both relieved let-7-mediated repression of R-luc protein and mRNA level (∼75% derepression at 24 hours after plasmid transfection; Fig. 3C, D). We then measured let-7-mediated deadenylation by LM-PAT (Fig. 3E; six hours after plasmid transfection). This revealed no significant change in R-luc-3xb mRNA deadenylation with either RCK or TNRC6A depletion, or for control siRNA transfection. Minor differences in LM-PAT data between conditions discernible in Fig. 3E were due to the dual transfection regimen, but no consistent trend emerged in three to four independent biological repeats (data not shown) nor did this variability ever approach the distinct tail length stabilization induced by let-7 anti-miR treatment. We furthermore transfected *in vitro* transcribed capped R-luc-mut mRNA with or without a poly(A) tail into knock-down cells (Fig. 3F; see below for details), to test whether we inadvertently impaired the poly(A) tail-dependence of cellular translation by depleting RCK or TNRC6A. This was not the case as each cellular condition gave similarly strong responses to the presence of a poly(A) tail (Fig. 3F).

RCK and GW182 both have reported roles in the miRNA mechanism [13], [14], [15], [46], [55], [56], [57], [58], [59]. Our finding that RCK depletion does not prevent let-7-mediated deadenylation is consistent with previous studies in *D. melanogaster* cells, where depletion of RCK/Me31B, together with other decapping activators, led to accumulation of deadenylated miRNA-target mRNAs [60]. Work on human and *D. melanogaster* GW182 proteins showed that their silencing activity could in part be attributed to mRNA deadenylation [16], [41]. However, GW182 proteins have repressive effects that are separable from their role in deadenylation and the situation in vertebrates is further complicated by ample opportunity for functional redundancy and specialisation among the three paralogs TNRC6A, B, and C [12], [13], [15], [42], [46]. Our observation that let-7-mediated deadenylation was unimpeded in cells depleted of TNRC6A may thus simply mean that we have not been able to reduce GW182 proteins below a required threshold level. With regard to the main objective of these experiments, we can nevertheless conclude that Ago co-factor depletion can cause significant relief of let-7-mediated repression despite ongoing target mRNA deadenylation, indicating that deadenylation alone is not sufficient to generate full repression.

### Presence of a poly(A) tail enhances let-7-mediated repression

**Figure 4:**
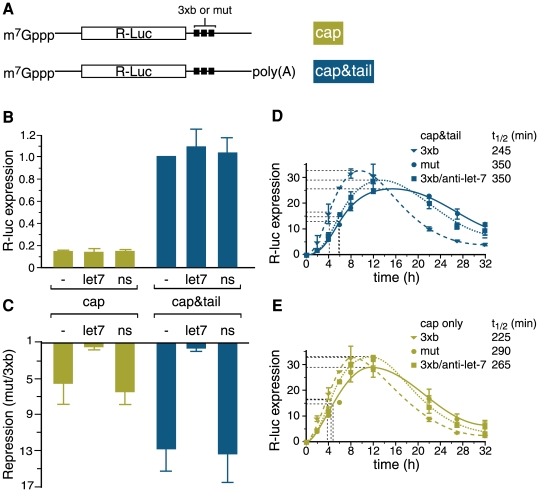
Full let-7-mediated translational repression of R-luc-3xb mRNA requires a poly(A) tail. HeLa cells were transfected with capped reporter mRNAs and incubated for 16 hours. (*A*) Schematic of the variant R-luc-3xb and mut mRNAs. (*B*) R-luc activity from the R-luc-mut mRNAs co transfected with LNAs indicated (normalized to F-luc reference; expression from the cap&tail mRNA is set to 1.0 for each experiment prior to averaging across experiments). (*C*) Repression by let-7 was calculated as in Fig. 1B. Averaged results from 4–7 independent triplicate experiments are shown with standard error in B and C. (*D, E*) Multiple cell aliquots were co-transfected with R-luc reporter mRNAs and LNAs as indicated. Cells were harvested at different times to measure R-luc protein expression, which was not normalized to F-luc. Three time series of each kind (with duplicate measurements) were each scaled to total level of recovered R-luc protein and then averaged. Error bars represent standard error (except at two [range] and six hours [no error bars]). The functional half-life of an mRNA in cells is defined as the time required for half-maximal accumulation of R-luc activity [19], [62].

Next we directly examined a possible contribution of the poly(A) tail and its removal to translational repression by let-7. To this end we employed a direct mRNA transfection approach [18], [19], [48]. We prepared m^7^GpppG-capped R-luc-3xb and R-luc-mut mRNA *in vitro* (Fig. 4A, Fig. S3) with or without a poly(A) tail and transfected these into HeLa cells together with an F-luc control. Measurement of reporter protein levels, normalisation for transfection efficiency and calculation of repression by let-7 was done as for the plasmid transfection experiments described above. This showed that adding a poly(A) tail to R-luc-mut mRNA improved translation as expected (five to six-fold over cap alone, measured 16 hours after mRNA transfection; Fig. 4B, see also Fig. 3F). Repression by let-7 was significantly stronger for the cap&tail form of R-luc mRNA (∼13-fold) than for capped mRNA (∼6-fold repression; Fig. 4C). (Similar differences in translational repression between cap and cap&tail forms of target mRNA were seen in undifferentiated P19 cells co-transfected with synthetic let-7 (Fig. S4)). To determine the contribution of let-7-mediated mRNA destablisation to repression, we measured R-luc mRNA levels recovered from transfected HeLa cells by real-time RT-PCR and found no significant differences between 3xb and mut mRNAs (data not shown). Given the possible complications with measuring physical mRNA stabilities in RNA-transfected cells [19], [61], we also estimated functional mRNA half lives [62], by following the accumulation of R-luc protein over time (Fig. 4D, E). This revealed that R-luc 3xb mRNA was moderately less stable than the mut control (∼1.4 fold for the cap&tail and ∼1.3 fold for the cap only versions), confirming previous observations that let-7 mediated repression of transfected R-luc mRNA occurs mainly at the level of translation [18]. Collectively, these data demonstrate that the presence of a poly(A) tail on a target mRNA can increase the magnitude of let-7-mediated translational repression. These results mirror our previous observations with the miCXCR4 reporter system [19], but they are counter to previous observations that the poly(A) tail did not contribute to repression of R-luc-3xb mRNA by let-7 [18]. Normalization to the parental RL mRNA instead of R-luc-mut (Fig. S5 and Supporting Information S1) may have masked a poly(A) tail contribution to let-7-mediated repression in previous work [18].

### A block to let-7-mediated deadenylation impairs translational repression

**Figure 5:**
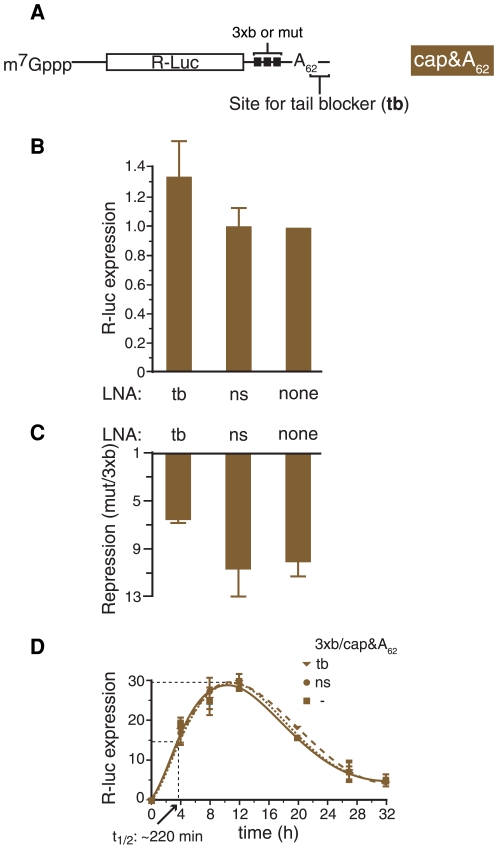
Blocking mRNA deadenylation impairs let-7-mediated translational repression. (*A*) Schematic of the R-luc 3xb and mut cap&A_62_ mRNAs indicating the site of ‘tail blocker’ (tb) binding. (*B, C*) HeLa cells were transfected with capped reporter mRNAs and incubated for 16 hours. (*B*) R-luc activity from the R-luc-mut-A_62_ mRNAs cotransfected with tb or control (ns) LNA or no LNA (none). Normalized expression without LNA is set to 1.0. (*C*) Repression by let-7 was calculated as in Fig. 1. Averaged results from 4–7 independent triplicate experiments are shown with standard error in E and F. (*D*) Multiple cell aliquots were co-transfected with R-luc reporter mRNAs and LNAs as indicated (as for Fig. 4D,E). Cells were harvested at different times to measure R-luc protein expression, which was not normalized to F-luc. Two time series of this kind (with duplicate measurements) were each scaled to total level of recovered R-luc protein and then averaged. Error bars represent data range.

Finally, we devised a strategy to specifically interfere with poly(A) tail removal, which involved sterically blocking the 3′ end of the target mRNA's poly(A) tail. To achieve this, we prepared capped R-Luc (3xb or mut) mRNAs carrying a poly(A) tail of defined length (cap& Fig. 5A). These mRNA were transcribed *in vitro* from plasmids that were modified to include an A_62_ segment downstream of the let-7 target sites to template the poly(A) tail (see Materials & Methods for plasmid details). The plasmid templates were linearised just downstream of this segment so that the A_62_ tail of the resulting mRNAs was followed by an 8 nt heteropolymeric 3′ tag. We first ascertained that this A_62_ tail still strongly stimulated translation, by comparison of cap&A_62_ with cap only versions of the R-luc-mut mRNA after transfection into HeLa cells (data not shown). We also found that appending the A_62_ tail and 8 nt 3′ tag instead of a longer poly(A) tail to the R-luc reporter mRNAs by itself did not affect on let-7-mediated repression, which was still strong (∼10.5 fold repression, measured 16 hours after mRNA transfection, Fig. 5C). This suggested that there is little/no variance in the mechanism of miRNA action in the presence of the 8 nt tag 3′ of the poly(A) tail.

To block access of deadenylases to the mRNA 3′ end, we co-transfected HeLa cells with R-luc-(3xb or mut)-cap&A_62_ mRNA and the ‘tail-blocker’ (tb) LNA complementary to the 3′ tag or an unrelated control LNA (ns). We found that expression of R-luc protein from the R-Luc-mut mRNA tended to be mildly elevated in the presence of tb LNA compared to transfection of non-specific control LNA or mock transfection (Fig. 5B), consistent with a block of default deadenylation of this mRNA. Importantly, we observed that the tb LNA specifically reduced let-7-mediated repression to ∼6-fold (Fig. 5C). tb LNA co-transfection had no appreciable effect on either physical (data not shown) or functional stability (Fig. 5D) of R-Luc/cap&A_62_ mRNA, indicating that the tb LNA impaired repression at the level of translation. We were unable to measure the tail length dynamics of the R-luc cap&A_62_ mRNA in transfected cells as the 3′ tag interfered with the LM-PAT assay (data not shown). To demonstrate that the tb LNA prevented reporter mRNA deadenylation as intended, we incubated our R-luc mRNAs in HeLa cell extracts [63], [64], which we found capable of enacting let-7-mediated deadenylation. Using this system as a surrogate, we confirmed that the tb LNA protected the mRNA 3′ end (Fig. S6).

Altogether, these results show that removal of the poly(A) tail is not essential for miRNA-mediated translational repression. Nevertheless, they also indicate that deadenylation can augment miRNA-mediated translational repression in mammalian cells.

## Discussion

We show here using a reporter system in HeLa cells that let-7 triggers target mRNA deadenylation, which is evident before measureable translational repression and mRNA destabilisation. The presence of a poly(A) tail on target mRNA enhances repression, while blocking tail removal by deadenylation reduces the magnitude of translational repression. Nevertheless, let-7-mediated repression can be attenuated despite ongoing deadenylation as observed in cells depleted of RCK or TNRC6A. Thus deadenylation is not essential for, but can quantitatively contribute to, let-7-mediated translational repression. Our findings are also consistent with a role of deadenylation in miRNA-mediated mRNA decay. In this way, we concur with and extend similar conclusions reached in other systems as referred to throughout this paper. A distinguishing feature of the present study is its detailed description of the relationship between miRNA-mediated deadenylation and translational repression in mammalian cells.

mRNA polyadenylation control is a versatile means to regulate gene expression [47], [65] and mRNA deadenylation is emerging as a widespread feature of miRNA action. In zebrafish embryos, miR-430 facilitates the clearance of hundreds of maternal mRNA at the onset of zygotic transcription [38], and reporters encoding 3′ UTRs from several of these mRNAs were shown to be deadenylated in a miR-430-dependent manner [38], [39]. miR-125b or let-7 were shown to hasten deadenylation and decay of responsive reporters in mammalian cells [40]. *In vitro* systems to study miRNA-mediated translational repression of mammalian or *D. melanogaster* origin also feature miRNA-mediated deadenylation [25], [28]. Degradation of miRNA-sensitive 3′ UTR reporters was shown to involve the CCR4:NOT deadenylase and DCP1:DCP2 decapping complexes in *D. melanogaster* [41]. Microarray analyses demonstrated that 60% of mRNA stabilised in the absence of Ago 1 were also increased in cells depleted of two different CCR4:NOT subunits [42]. We show here that miRNA-mediated mRNA deadenylation is readily observable with both reporters and endogenous mRNAs in mammalian cells.

Deadenylation is the first step in canonical mRNA turnover [66] and it thus contributes to the decay-promoting activity of miRNA. Its relationship with miRNA-mediated translational repression has been less clear. Deadenylation could arise as a consequence of translational repression by a miRNA. This is a plausible hypothesis for which there is precedent [67]. However, it has been shown in zebrafish, mammalian and *D. melanogaster* cells that blocking translation initiation on reporter mRNAs by interfering with either 40S recruitment [39], [42], 5′ UTR scanning [40], or start codon recognition [38] does not prevent miRNA-mediated deadenylation. We add our observation that miRNA-mediated deadenylation is evident prior to the onset of measurable translational repression. Taken together, it is thus apparent across different systems that miRNAs can stimulate deadenylation without a specific requirement for mRNA translation or a prior translational repression step.

Alternatively, deadenylation may exclusively stimulate mRNA decay. This has been addressed in mammalian and *D. melanogaster* cells using reporter constructs designed to give rise to mRNAs either carrying the 3′ end stem-loop of histone mRNA instead of a tail, or lacking a tail due to the presence of a self-cleaving ribozyme in the 3′ UTR [40], [42], [46]. These mRNAs could still be silenced, although at times not as strongly as their polyadenylated counterparts. The common observation is thus that miRNAs can repress translation without a requirement for mRNA deadenylation. It does, however, not necessarily follow that deadenylation has no role in translational repression, as shown by our present findings and previous reports that translational repression is enhanced by the presence of a poly(A) tail [19], [20], [25], [28]. Most importantly, we show here that while blocking let-7-mediated deadenylation does not completely abolish let-7-mediated translational repression, it nevertheless clearly attenuated it. This is a direct demonstration in living cells that mRNA deadenylation can specifically contribute to translational repression by a miRNA.

Taken together with the extant literature, our data thus favours a model whereby mRNA deadenylation is a proximal miRNA action that not only promotes mRNA decay but also attenuates the stimulatory role of the poly(A) tail in mRNA translation, by generating an oligo-adenylated form of the mRNA that associates less with cytoplasmic PABP. Indeed, mammalian miRNA target mRNAs are enriched in Ago and GW182 immunoprecipitations, while they are underrepresented in complexes containing PABPC4 [12]. The oligo-adenylated mRNA is further silenced by co-repressors, which most plausibly interfere with cap/eIF4E function [18], [19], [20], [23], [24], [25], [26], [28], and then is either stably stored or destroyed by decapping and exonucleolytic degradation. The magnitude of translational repression due to miRNA-mediated deadenylation may depend on the extent to which the poly(A) tail contributed to target mRNA translation before removal; this will vary between mRNAs and with the functional state of the cellular translational machinery. For instance, cells may express variable amounts of several variants of cytoplasmic PABP [68], [69] and PABP activity is know to be regulated by specific inhibitor proteins such as PAIP2 [70]. Such differences may explain some of the divergent literature on miRNA-responses to the absence of a poly(A) tail on target mRNA. The balance between decay and storage of target mRNA may further be determined through interplay between the specific (3′ UTR) sequence context and the given cellular environment. With these provisos, the model explains most reports assessing the role of the poly(A) tail in miRNA-mediated repression.

The above scenario clearly does not accommodate post-initiation effects by miRNAs, and several reports have further presented experimental evidence or theoretical arguments to link miRNA-mediated repression to late sub-steps of translation initiation [22], [71], [72], [73]. More work will have to be done to address the possibility that miRNAs affect translation at more than one step. The poly(A) tail also stimulates the 60S subunit joining step during late initiation and it is linked to translation termination through an interaction between PABP and eRF3 [44], providing ways to rationalise a role for deadenylation in several alternative models of translational repression by miRNAs.

## Materials and Methods

### DNA constructs, siRNAs and LNA oligonucleotides

#### DNA constructs

The plasmids pRL, pRL-3xbulge and pRL-3xbulgemut, which encode Renilla luciferase driven from a CMV promoter, were gifts from W. Fillipowicz (Friedrich Miescher Institute for Biomedical Research, Basel, Switzerland) [18]. The three let-7 binding sites in R-luc-3xb are engineered ∼10 nt downstream of the stop codon. Plasmid transfection into cells results in production of R-luc mRNAs with a 3′UTR ∼260 nt in length and the poly(A) tail added at an SV40 late poly(A) signal ∼160 nt from the last let-7 binding site. R-luc-3xb/mut mRNAs transcribed from these plasmids *in vitro* differ slightly in that transcription begins at the T7 promoter and the poly(A) tail is added at the Hpa1 site 7 nt preceding the SV40 poly(A) signal cleavage site. The Firefly luciferase pGL3 plasmid was obtained from Promega. The pRL-A_62_ constructs (Fig. 5) were engineered from pRL-3xb and pRL-mut backbones. Briefly, this involved inserting a Not I fragment from pFL-WT [23] (containing six miR-2 binding sites and a stretch of 62 adenosines) into the Not I site of the pRL-3xb and pRL-mut constructs (within the R-luc 3′UTR). The six miR-2 binding sites were then removed by digestion with SacII and re-ligation of the remaining backbone, creating the pRL-A_62_ constructs. Linearisation of this pRL-pA_62_ construct with EcoICRI enzyme (Promega) creates an eight nucleotide overhang after the poly(A) tail stretch. The *in vitro* transcribed transcripts of RL-3xb/mut-cap&A_62_ mRNA encode a 3′UTR of ∼107 nt, with the encoded poly(A)_62_ starting 20 nt downstream of the last let-7 miRNA binding site.

#### Locked nucleic acid (LNA) oligonucleotides

LNA modified anti-miR oligonucleotides were obtained from Exiqon (MA, USA) with the following sequences: hsa-let-7a, AACTATACAACCTACTACCTCA, hsa-miR-499, TTAAACATCACTGCAAGTCTTAA. LNA used with the R-luc-3xb-A_62_ and R-luc-mut-A_62_ mRNAs have 100% LNA chemistry with the following sequences: Tail blocker (tb), CTCGGTACTTTT, complementary to the tag sequence 3′ of the A_62_ segment; non-specific (ns) control, CGCTGTATTTTT.

#### Synthetic siRNA/miRNAs

Synthetic duplex miRNA sense sequences used (Fig. S4) were: let-7a UGAGGUAGUAGGUUGUAUAG UU and miCXCR4, UGUUAGCUGGAGUGAAAAC UU [74], [75]. siRNA sense sequences used in the depletion studies (Fig. 3) were GW182 (TNRC6A), GAAAUGCUCUGGUCCGCUA [55], [57], [58] and RCK, GCAGAAACCCUAUGAGAUU UU [55]. miCXCR4 was used in these experiments as the non-specific (ns) control.

### *In vitro* transcription of mRNA

pRL-3xbulge and pRL-3xbulgemut were linearized with Hpa1 and pRL-A_62_-3xbulge and pRL-A_62_-3xbulgemut were linearized with EcoICRI (Promega). DNA was purified by phenol/chloroform extraction and ethanol precipitation and used as template for *in vitro* transcription reactions as described [19]. mRNAs were purified using either the NucAway kit (Ambion) followed by two phenol/chloroform extractions and ethanol precipitation, or the Megaclear kit (Ambion). Concentrations were estimated by A_260_ and mRNA quality inspected using the RNA 6000 Nanochip kit on an Agilent 2100 bioanalyzer.

### Cell culture, transfections and Dual luciferase assays

HeLa cells were maintained in DMEM with 5% FCS, supplemented with glutamine and penicillin/streptomycin, as detailed in [19], [48]. Mouse embryonal carcinoma P19 cells were maintained in the same media and induced to differentiate as described [76]. Growth conditions for SH-SY5Y cells (Fig. S1) were as previously reported [77].

#### mRNA transfections

For mRNA transfections, cells were seeded in a 24-well plate and transfections were performed in triplicate at ∼60–70% confluency by adding a preincubated (30min, 25°C) transfection solution (200 µl) and 300 µl of Opti-MEM I (Invitrogen) to each well. The 200 µl of transfection solution (in Opti-MEM I) contained 1 µl Lipofectamine 2000 (Invitrogen), 10 or 20 ng Renilla luciferase-encoding (R-luc) mRNA and 80 ng pGL3 (F-luc control). Cells were harvested 16 hours after transfection unless stated otherwise. For anti-miR LNA transfections (Fig. 1), 200 µl of transfection solution also contained 5 pmoles of LNA. For the tail blocking experiments (Fig. 5), 1.6 pmoles of tail blocking (tb) or non-specific (ns) LNA was added to the transfection solution. For depletion studies (Fig. 3), siRNA was transfected into cells at ∼35–50% confluency 30 hours prior to mRNA transfection. To transfect one well, 100 µl of transfection solution (in Opti-Mem I) contained 10 pmole siRNA and 1 µl RNAiMAX Lipofectamine (Invitrogen) and was transfected into cells according to the manufacturer's instructions. 4–8 hours after transfection the medium was replaced with normal growth medium. Subsequent mRNA transfections were as described above except the 200 µl of mRNA transfection solution was added to 300 µl of normal growth medium (not Opti-Mem I). When using synthetic miRNA (Fig. S4) the transfection solution additionally contained 1 pmole of duplex miRNA.

#### Plasmid transfections

Plasmid transfections were performed similarly to mRNA, with the 200 µl transfection solution containing 30 ng of R-luc encoding plasmid and 600 ng of pGL3 F-luc. Medium was replaced ∼8 hours after transfection and cells were harvested at times specified. Additionally, in the time-course described in Fig. 2 and Fig. S2, cells were trypsinised and diluted two-fold to maintain an actively growing culture. For anti-miR LNA transfections (Fig. 1B, D, Fig. 4), 200 µl of plasmid transfection solution also contained 5 pmoles of LNA. For the depletion studies (Fig. 3), siRNA was transfected into cells 24 hours prior to plasmid transfections as described above for mRNA transfections.

#### P19 cell differentiation

For Fig. 1E, P19 cells were induced to differentiate in 500 nM retinoic acid (RA) on 15 cm^2^ bacterial grade plates for 48 hours before replating on fresh bacterial grade plates with fresh media plus RA for an additional 48 hours in RA to allow aggregates to form. Aggregates were plated out onto tissue culture grade plastic in the absence of RA on Day 4. Cells were harvested at the indicated time points [76].

#### Luciferase Reporter assays

These were performed using the Dual-Luciferase Reporter Assay system (Promega) in a FLUOstar Optima plate reader (BMG Labtech) as described [19].

#### Quantitative Western blotting

SDS-PAGE gels were transferred to Immobilon-FL membrane (Millipore). Anti-RCK (MBL International) and α-Tubulin antibodies (Santa Cruz) were used at 1∶1000 working dilution. Fluorescent secondary antibodies were anti-rabbit/alexa-750 and anti-mouse/alexa-680 conjugated antibodies (Invitrogen). Blot hybridisation and detection was performed using the Odyssey Infrared Imaging System and buffers according to the manufacturer's instructions (*LI-COR* Biosciences).

### RNA analyses

#### Purification of total RNA from cells in culture

This was done using Trizol (Invitrogen) according to the manufacturer's instructions.

#### Quantitative reverse transcriptase PCR

For analysis of reporter mRNA levels (Figs. 1B, 2, 3), HeLa cells were transfected in 12-well plates by scaling up the plasmid transfection protocol detailed above. Before RNA purification, cells were trypsinised and washed in PBS to remove unincorporated transfection mixtures. Purified total RNA was then treated with Turbo DNAse (Ambion) as per the manufacturer's instructions. 500-1000 ng of RNA was reverse-transcribed with Superscript III reverse transcriptase (Invitrogen) using a random hexamer primer, as per manufacturer's instructions, while a no-RT control was set up in parallel to assess the efficiency of the DNAse treatment. mRNA levels were assessed by Quantitative (q)PCR on a Lightcycler 480 using Lightcycler 480 SYBR green 1 Master reagent (Roche) as per manufacturer's instructions. Primers used to measure reporter mRNA levels are described in Table S1. R-luc mRNA levels were normalised to F-luc mRNA levels where indicated.

For qPCR analysis of siRNA-mediated depletion efficiencies (Fig. 3A) *RCK* and *TNRC6A* mRNA levels were normalised to mRNA levels of the ribosomal protein *RPL13a*. Primer sequences are shown in Table S1.

#### Oligo-mediated RNase H cleavage and high resolution northern blot

Cleavage reactions were performed as previously described (http://www.mcb.arizona.edu/parker/PROTOCOLS/protocols.htm), except that RNase digestion was for 45 min at 37°C. The cleavage oligonucleotides used in this study were RH-R-Luc CTTCAGCACGCGCTCCAC and RH-Hs GAPDH CTTGCTGGGGCTGGTGGTCC. RNA (5 µg total cellular RNA, Fig. 1C, or 25–50% of RNA recovered from *in vitro* deadenylation assays, Fig. S6) was separated by gel electrophoresis using 5% polyacrylamide 7M urea denaturing gels, then transferred onto onto GeneScreen Plus membrane (PerkinElmer). Membranes were crosslinked (240 mJ) using a UV Stratalinker 2400 (Stratagene). Probe labelling and hybridisation was as described for the miRNA northern blot. The probe to detect GAPDH in Fig. 1C (right panel) was a ^32^P-labelled TVN-GAPDH PCR product, labelled with ^32^P-CTP by random priming (see below).

#### Ligation-mediated poly(A) test

To measure poly(A) tail lengths of multiple individual mRNAs, we used the Ligation-Mediated Poly(A) Test (LM-PAT) assay as described [47], [48], [78]. The anchor primer used in the RT step and common reverse PCR primer is GCG AGC TCC GCG GCC GCG TTT TTT TTT TTT. The (dT)_12_VN primer used in the RT to generate a size marker for the shortest possible LM-PAT product was, GCG AGC TCC GCG GCC GCG TTT TTT TTT TTT VN [78]. Gene-specific primers are shown in Table S1. For every mRNA we analysed, LM-PAT products were excised from the agarose gel, sub-cloned and sequenced to ensure that we obtained genuine amplification products. Visualisation of LM-PAT products at early times after plasmid transfection (Fig. 2C) was performed by Southern blotting. PCR products were transferred to a nitrocellulose membrane and the probe for Southern blotting was a ^32^P-labelled TVN-R-luc PCR product, labelled with ^32^P-CTP by random priming. Detection of LM-PAT products and blots was by fluorescence scans or phosphorimaging using an FLA-5100 imager and MULTIGAUGE software (Fujifilm).

#### miRNA northern blot

To measure let-7 expression in P19 and HeLa cells (Fig. 1E, Fig. S4A), total RNA was separated by gel electrophoresis using 4% stacking/12% resolving polyacrylamide/7M urea denaturing gels, then transferred onto GeneScreen Plus membrane (PerkinElmer). Membranes were crosslinked (120 mJ) using a UV Stratalinker 2400 (Stratagene). Probes (let-7a anti-sense probe was AACTATACAACCTACTACCTCA, see above for U6 probe) were ^32^P end-labelled using Polynucleotide kinase (NEB) according to the manufacturer's instructions, and incubated with the membrane in hybridization buffer (250 mM NaPO4, pH 7, 7% SDS) at 42°C, then washed twice with 2xSSC. Detection by phosphorimaging used an FLA-5100 imager and MULTIGAUGE software (Fujifilm).

## Supporting Information

Supporting Information S1 Supporting Results and Methods
(0.10 MB DOC)

Figure S1 Endogenous miRNA targets commonly exhibit short poly(A) tails. LM-PAT assays of miRNA targets E2F5, MYO10 and VAMP3 mRNA (SH-SY5Y cells) as well as RAVER, DNAJB11 and SERBP1 (HeLa cells). ACTB mRNA served as control.
(1.07 MB EPS)

Figure S2 Timecourse of protein and mRNA expression in transiently transfected HeLa cells. HeLa cells were cotransfected with either pRL-3xbulge or pRL-3xbulgemut plasmid [8] and pGL3 (F-luc control). Protein and RNA was harvested from cells 1–48 hours after plasmid transfection. Cells were trypsinised and diluted two-fold at 24 hours to maintain actively growing cultures. (A) Raw R-luc activity (prior to normalisation). Points represent average of triplicates with standard error. (B) R-luc mRNA levels measured by qPCR and normalised to total input RNA. Points represent average of triplicates with standard error. These values were further used to calculate repression as depicted in Fig. 2A. To this end, R-luc levels (mRNA or protein) at each time point were further normalised to the F-luc transfection control. No repression was calculated for the one-hour time point as values were near background.
(0.66 MB EPS)

Figure S3 Microfluidic assessment of in vitro transcribed mRNAs. (A) Schematic of the variant R-luc mRNAs. (B) Variant R-luc-3xb and mut mRNA, as well as parental R-luc mRNA, were transcribed in vitro and analysed by microfluidics.
(0.82 MB EPS)

Figure S4 The mRNA poly(A) tail contributes to translational repression by synthetic let-7a. (A) Endogenous expression of let-7 in HeLa cells and P19 cells, either undifferentiated (−) or differentiated by retinoic acid treatment (RA), was measured by northern blotting (100 µg total RNA loaded per lane). (B, C) Undifferentiated P19 cells were co-transfected with R-luc mRNA (3xb or mut), pGL3 (F-luc control) and synthetic let-7 or control miRNA followed by incubation for 16 hours. (B) R-luc activity from the R-luc-mut mRNAs transfected into P19 cells (normalised to F-luc reference; expression from the cap&tail mRNA is set to 1.0). (C) Repression by synthetic let-7a in P19 cells was calculated by dividing the normalized R-luc activity from cells co-transfected with the miCXCR4 control by the normalized R-luc activity from cells co-transfected with let-7a (filled bars: R-luc-3xb mRNA, open bars: R-luc-mut mRNA). Averaged results from 4-7 independent triplicate repeat experiments are shown with standard error in B and C.
(1.71 MB EPS)

Figure S5 Normalisation to R-luc-RL mRNA can mask the poly(A) tail dependence of miRNA-mediated repression. (A,B) HeLa cells were co-transfected with R-luc mRNA (3xb or RL) and pGL3 (F-luc control) and incubated for 16 hours. (A) R-luc activity from the R-luc-RL mRNAs transfected into HeLa cells (normalised to F-luc reference; expression from the cap&tail mRNA is set to 1.0). (B) Repression by endogenous let-7 in HeLa cells. Repression was calculated as for Fig. 1. Averaged results from 5 independent triplicate repeat experiments are shown with standard error.
(0.49 MB EPS)

Figure S6 The tb LNA blocks let-7-mediated mRNA deadenylation in vitro. (A) Schematic of the R-luc 3xb and mut cap&A62 mRNAs indicating the site for oligonucleotide-mediated RNase H cleavage. (B) R-luc 3xb or mut cap&A62 mRNAs were incubated in cell-free translation reactions based on HeLa extract for 30–180 min as indicated. Recovered RNA was subjected to RH-R-Luc oligonucleotide-mediated cleavage by RNAse H and northern blot analysis as in Fig. 1C. Labels to the left of the panel mark the position of the 3′ UTR fragments carrying different lengths of poly(A) tail. (C) As in B, except that reactions additionally contained anti-miR LNAs as indicated. Reactions were incubated for 120 min. Control cleavage reactions with input R-luc 3xb cap&A62 mRNA in the presence or absence of oligo(dT) generate 3′ UTR fragments with or without intact poly(A) tail, shown on the right. (D) As in C, except that R-luc 3xb cap&A62 mRNA was annealed to specific (tb) or control (ns) LNA prior to incubation. Control cleavage reactions are shown on the left; probing for U6 snRNA as a loading control is shown in the lower panel.
(16.18 MB EPS)

Table S1 Gene specific primer sequences for quantitative PCR and LM-PAT assays.
(0.04 MB DOC)
