# Supplementary material for: microRNA-Mediated Messenger RNA Deadenylation Contributes to Translational Repression in Mammalian Cells
Source: PLoS One. 2009 Aug 27;4(8):e6783. doi: 10.1371/journal.pone.0006783 (PMC2728509; doi:10.1371/journal.pone.0006783)
Supplement: Supporting Information S1 — Supporting Results and Methods (0.10 MB DOC) [file pone.0006783.s001.doc]

SUPPORTING INFORMATION S1

Supporting Results

*Characteristics of the LM-PAT assay.* To measure poly(A) tail lengths of mRNAs, we applied the ligation-mediated poly(A) test (LM-PAT; schematic in Fig. 1C). This RT-PCR-based assay yields product sizes that reflect the poly(A) tail lengths present on a specific mRNA [1,2,3]. The assay is sensitive and can be readily applied to any mRNA of choice. We validated the assay using the abundant GAPDH mRNA as an example and it yielded results equivalent to high resolution northern blotting (Fig. 1C). By contrast, northern blotting was not sensitive enough to detect R-luc reporter mRNA tail length in our transfection regimen (data not shown; but see Fig. S6). In the LM-PAT assay RNA is first incubated with oligo(dT)_12–18_ primers in the presence of T4-DNA ligase at 42°C. This creates a poly(dT) copy of each mRNA's poly(A) tail within the sample. Addition of an excess of anchor-(dT)_12_ primer and further incubation at 12°C favours annealing of the anchor primer to unpaired poly(A) ends and ligation to the poly(dT) stretch. This assembly is used to prime synthesis of first-strand cDNA by reverse transcription. Aliquots of this cDNA are then used as templates in PCR reactions with primers specific to the mRNA 3'-UTR of choice and the anchor region. To generate a size marker for the shortest possible LM-PAT product, cDNA is additionally synthesised with an anchor-(dT)_12_VN primer to clamp the anchor to the 3’UTR–poly(A) junction [3]. PCR-products generated from this ‘clamped’ cDNA library are labelled ‘TVN’ in the Figures. Note that the assay principle introduces some ‘laddering’ of PCR products. Specifically, the lowest ‘rung’ in the LM-PAT ladder (labelled ~12 in the Figures) potentially represents any short tails (~7-22 adenosines) that can accommodate a single anchor–(dT)_12_ but no additional oligo(dT)_12-18_ primer. For every mRNA we analysed, LM-PAT products were excised from the agarose gel, sub-cloned and sequenced to ensure that we obtained genuine amplification products. We have previously published detailed analyses of LM-PAT assay performance [1,2].

*Propensity of miRNA targets to accumulate with short poly(A) tails at steady-state.* The steady-state poly(A) tail length of several validated miRNA targets was assessed in cell lines reported to express both the mRNAs and cognate miRNAs. We focussed on mRNAs that were identified in Ago/RNP immuno-purifications identified by two separate groups. From the first study [4], we monitored steady-state adenylation of *E2F5*, *MYO10* and *VAMP3*, validated as targets of miR-124a that are not significantly destabilised by miRNA-mediated repression. The human neuroblastoma cell line SH-SY5Y expresses both miR-124a [5] and the mRNAs in question. Based on the second study [6], we chose four validated miRNA-targeted mRNAs, *Raver2*, *DNAJB11*, *SERBP1* and *HMGB1*. HeLa cells express the miRNAs [5] that have been identified as regulators of these mRNAs (*Raver2*, miR-99a, miR-99b and let-7a; *DNAJB11*, miR-29b; *SERBP1*, miR-26a and miR-103; and *HMGB1*, miR-141 [albeit only at low level]). With the exception of *HMGB1* (data not shown), all the miRNA-targeted mRNAs accumulated shortened poly(A) tails at steady-state of a size equivalent to that of the TVN marker for oligo-adenylated forms (Fig. S1). Additionally, we have observed predominantly oligo-adenylated forms at steady-state for a further eleven of twelve endogenous mRNAs, which had been implicated as miRNA targets in ways other than by co-purification with an Ago-RNP (data not shown). Oligo-adenylated forms do not accumulate with our control mRNAs *ACTB* or *GAPDH*, which were not identified as miRNA targets through Ago co-purification [4,6], nor do they have strongly predicted miRNA-binding sites in their 3’UTRs. Although we did not pursue most of these observations further by LNA anti-miR transfection, these data nevertheless suggest that miRNAs commonly promote target mRNA deadenylation in mammalian cells, similar to the situation in *D. melanogaster* cells [7].

*Differences in apparent poly(A) tail-dependence of let-7-mediated translational repression due to choice of reference mRNA.* To reconcile our findings with previous observations, we performed analyses of let-7-mediated repression of R-luc with or without a poly(A) tail using R-luc mRNAs transcribed from the parent vector pRL as the unrepressed control [8] instead of R-luc-3xmut mRNA. We noted that a poly(A) tail only marginally increased translational output of RL mRNA in HeLa cells (~1.5-fold, Fig. S5B). Consequently, normalisation to the RL control (instead of R-luc-mut) largely masks the contribution of the poly(A) tail to let-7-mediated repression (Fig. S5C).

*In vitro tests of the efficacy of the ‘tail blocker’ LNA*. To directly demonstrate that terminally annealed tail blocker (tb) LNA can block mRNA deadenylation we turned to a cell-free translation system based on HeLa cell extracts [9,10]. Incubating R-luc 3xb and mut reporter mRNAs in this system for up to 3 hours we found that it did not recapitulate let-7-mediated R-luc 3xb mRNA translational repression to an appreciable extent (data not shown), echoing previous observations with HEK293F extracts that only showed miRNA-mediated repression when derived from cells overexpressing miRNA pathway components [11]. This, as well as other published works using *in vitro* translation systems [11,12,13,14] suggest several options that could be tried to make this a fully operational *in vitro* system to study miRNA action. Using the cell-free system it was, nevertheless, readily possible to test for deadenylation of the R-luc mRNA by oligonucleotide-mediated RNase H cleavage and high resolution Northern blotting (Fig. S6A). This revealed a preferential, time-dependent deadenylation of R-luc 3xb cap&A_62_ over R-luc mut cap&A_62_ mRNA (Fig. S6B). These observations also demonstrate that the 8nt 3’ tag alone is not sufficient to inhibit deadenylation. Furthermore, mRNA deadenylation led to the accumulation of a detectable intermediate exhibiting a residual tail of ~20 adenosines, which is consistent with the tail length seen on miRNA-targeted mRNAs in living cells by LM-PAT assay (the PCR product labelled ~12 in the Figures reports on tails ~7-22 adenosines in length). Notably, R-luc 3xb cap&A_62_ mRNA deadenylation *in vitro* was blocked by adding anti-let-7 LNA to the extract but not by adding the irrelevant anti-miR-499 LNA (Fig. S5C). Thus, the HeLa extract-based translation reactions recapitulate miRNA-mediated deadenylation *in vitro*. Next, we annealed R-luc 3xb cap&A_62_ mRNA to either the specific tb LNA or an unrelated control LNA (ns) and incubated the mRNA in the translation system. The tb LNA selectively protected the R-luc 3xb cap&A_62_ mRNA from generalised mRNA decay in the system (Fig. S6D; similar effects were seen with R-luc mut cap&A_62_ mRNA, data not shown). Importantly, the protected mRNA retained its full poly(A) tail, directly demonstrating that the ‘tail blocker’ strategy is efficacious in protecting the mRNA 3’ end.

**Supporting Materials and Methods**

*In vitro deadenylation assay (Fig. S6)*: HeLa cell extracts were prepared and *in vitro* translation reactions assembled essentially as previously published [9,10]. In brief, multiple translation reactions were assembled on ice, added to tubes containing 2.5 ng of R-luc mut or 3xb cap&A_62_ mRNA (10 µl final reaction volume), and immediately transferred to 37°C. Samples were removed at indicated time-points and snap frozen. 0.3 pmol anti-miR LNA oligonucleotides were added to some reactions. Where indicated, tail-blocking LNA (or non-specific control LNA) was pre-annealed to R-luc 3xb cap&A_62_ mRNA at a 100-fold molar excess in translation permissive buffer [16mM Hepes pH 7.4; 2mM Mg(OAc)_2_; 70mM K(OAc)]. The annealing reaction was heated to 70ºC for 5 min then cooled over a 20 min period to 37°C and snap frozen. Aliquots of these annealed hybrids were added to reaction mixes as described above. RNA was recovered by phenol/chloroform extraction and ethanol precipitation. As a precipitation aid, 10-20 µg of yeast total RNA were spiked in per reaction. The oligonucleotides used as probes were GGTAGTTCAATAGGCTGTGC (for R-luc) and GTGCTGCCGAAGCGATCAC (for U6 snRNA).

References for Supporting Information S1

1. Clancy JL, Nousch M, Humphreys DT, Westman BJ, Beilharz TH, et al. (2007) Methods to analyze microRNA-mediated control of mRNA translation. Methods Enzymol 431: 83-111.

2. Beilharz TH, Preiss T (2007) Widespread use of poly(A) tail length control to accentuate expression of the yeast transcriptome. RNA 13: 982-997.

3. Beilharz TH, Preiss T (2009) Transcriptome-wide measurement of mRNA polyadenylation state. Methods 48: 294-300.

4. Karginov FV, Conaco C, Xuan Z, Schmidt BH, Parker JS, et al. (2007) A biochemical approach to identifying microRNA targets. Proc Natl Acad Sci U S A 104: 19291-19296.

5. Landgraf P, Rusu M, Sheridan R, Sewer A, Iovino N, et al. (2007) A mammalian microRNA expression atlas based on small RNA library sequencing. Cell 129: 1401-1414.

6. Beitzinger M, Peters L, Zhu JY, Kremmer E, Meister G (2007) Identification of human microRNA targets from isolated argonaute protein complexes. RNA Biol 4: 76-84.

7. Eulalio A, Huntzinger E, Nishihara T, Rehwinkel J, Fauser M, et al. (2009) Deadenylation is a widespread effect of miRNA regulation. RNA 15: 21-32.

8. Pillai RS, Bhattacharyya SN, Artus CG, Zoller T, Cougot N, et al. (2005) Inhibition of Translational Initiation by let-7 MicroRNA in Human Cells. Science 309: 1573-1576.

9. Bergamini G, Preiss T, Hentze MW (2000) Picornavirus IRESes and the poly(A) tail jointly promote cap- independent translation in a mammalian cell-free system. RNA 6: 1781-1790.

10. Westman B, Beeren L, Grudzien E, Stepinski J, Worch R, et al. (2005) The antiviral drug ribavirin does not mimic the 7-methylguanosine moiety of the mRNA cap structure in vitro. RNA 11: 1505-1513.

11. Wakiyama M, Takimoto K, Ohara O, Yokoyama S (2007) Let-7 microRNA-mediated mRNA deadenylation and translational repression in a mammalian cell-free system. Genes Dev 21: 1857-1862.

12. Thermann R, Hentze MW (2007) Drosophila miR2 induces pseudo-polysomes and inhibits translation initiation. Nature 447: 875-878.

13. Mathonnet G, Fabian MR, Svitkin YV, Parsyan A, Huck L, et al. (2007) MicroRNA inhibition of translation initiation in vitro by targeting the cap-binding complex eIF4F. Science 317: 1764-1767.

14. Wang B, Love TM, Call ME, Doench JG, Novina CD (2006) Recapitulation of Short RNA-Directed Translational Gene Silencing In Vitro. Mol Cell 22: 553-560.

15. Vandesompele J, De Preter K, Pattyn F, Poppe B, Van Roy N, et al. (2002) Accurate normalization of real-time quantitative RT-PCR data by geometric averaging of multiple internal control genes. Genome Biol 3: RESEARCH0034.
