# Supplementary material for: microRNA-Mediated Messenger RNA Deadenylation Contributes to Translational Repression in Mammalian Cells
Source: PLoS One. 2009 Aug 27;4(8):e6783. doi: 10.1371/journal.pone.0006783 (PMC2728509; doi:10.1371/journal.pone.0006783)
Supplement: Table S1 — Gene specific primer sequences for quantitative PCR and LM-PAT assays. (0.04 MB DOC) [file pone.0006783.s008.doc]

| Name | Sequence |
| --- | --- |
| **qPCR** |  |
| Rluc for | GGC GAG AAA ATG GTG CTT GAG |
| Rluc rev | TCC TTG AAT GGC TCC AGG TAG G |
| Fluc for | TAC TGG TCT GCC TAA AGG TGT CG |
| Fluc rev | AAC CGT GAT GGA ATG GAA CAA C |
| RPL13a for | CCT GGA GGA GAA GAG GAA AGA GA |
| RPL13a rev [15] | TTG AGG ACC TCT GTG TAT TTG TCA A |
| RCK for | GGT GCC TAC CTC ATT CCC TTA C |
| RCK rev | ATG TGT TTG CTG ACC TGG ATG |
| TNRC6A for | AGA AGG GAA AGG GGA ATG |
| TNRC6A rev | TCC TCC AGA AAG GTC CAT C |
| **LM-PAT primers** |  |
| R-luc-PAT | GGC CGC TTC CCT TTA GTG AG |
| H.s GAPDH-PAT | GGA CCA CCA GCC CCA GCA AG |
| H.s HMGA2-PAT | CGC TTG CTT GTT GAA AAT ATT TCT CTA G |
| H.s ACTB-PAT | GAG CCT TCG TGC CCC CCC TTC C |
| H.s E2F5-PAT | GAC TTC TGA CAT TCC ACT TTC C |
| H.s MYO10-PAT | GCC AAC TAA GTC TAC CCA CAC G |
| H.s VAMP3-PAT | CCA CTG GCT CCT GCA TTA ACC C |
| H.s SERBP1-PAT | CGG TTT TGG TTA ACT TGG TTC |
| H.s DNAJB11-PAT | CCC TGG AGG CCA GTC CAG TGC |
| H.s RAVER2-PAT | GGC CTG TGC CCC GTT ATC AAT C |
| M.m GAPDH-PAT | CAG CAA CAG GGT GAT GGA CC |
| M.m HMGA2-PAT | GCA CAA TAA ACA TAA CAG CCT CTG TG |
